# Body image as a mediator in the relationship between psychotic experiences and later disordered eating: A 12-month longitudinal study in high school adolescents

**DOI:** 10.1192/j.eurpsy.2024.1164

**Published:** 2024-08-27

**Authors:** L. Houissa, S. Hallit, M. Cheour, A. A. Loch, F. Fekih-Romdhane

**Affiliations:** ^1^The Tunisian Center of Early Intervention in Psychosis, Department of Psychiatry “Ibn Omrane”, Razi hospital, Manouba; ^2^Tunis El Manar University, Faculty of Medicine of Tunis, Tunis, Tunisia; ^3^School of Medicine and Medical Sciences, Holy Spirit University of Kaslik, Jounieh, Lebanon; ^4^Psychology Department, College of Humanities, Effat University, Jeddah, Saudi Arabia; ^5^Applied Science Research Center, Applied Science Private University, Amman, Jordan; ^6^Research Department, Psychiatric Hospital of the Cross, Jal Eddib, Lebanon; ^7^Laboratorio de Neurociencias (LIM 27), Instituto de Psiquiatria, Hospital das Clinicas HCFMUSP, Faculdade de Medicina, Universidade de Sao Paulo; ^8^Instituto Nacional de Biomarcadores em Neuropsiquiatria (INBION), Conselho Nacional de Desenvolvimento Cientifico e Tecnológico, Sao Paulo, Brazil

## Abstract

**Introduction:**

Psychotic experiences (PE) and disordered eating (DE) are frequently observed among the general population, especially in childhood and adolescence. However, the relationship between the two groups of disorders is still unclear.

**Objectives:**

To explore the hypothesis that the pathways from PEs to DE are mediated by body-image disturbances in a sample of adolescents

**Methods:**

We conducted a 12-month longitudinal study on high school students from four different high schools from the Ariana governorate, from April 2022 to April 2023.

Participants were evaluated at baseline then every 6 months with a target length of follow-up of 1 year.

The questionnaire contained

Questions about socio demographic variables

The Eating Attitude Test (EAT-26)

The Multidimensionnal Body Self-Relations Questionnaire Appearance Scale (MBSRQ-AS)

The Community Assesment of Psychic Experiences (CAPE-42)

**Results:**

**1) Sample characteristics**

Sample was constituted of 510 individuals. Of those, 312 (61.2%) were females. Mean age was of 16.05 (SD=1.01) years.

The majority of the students resided in urban areas, accounting for 97.8% of the total.

When it comes to family income, 4.1% of the students’ families had an income of less than 1000 Tunisian Dinars (TD), 25.9% had an income between 1000 and 2000 TD, 32.2% had an income ranging from 2000 to 3000 TD, and the remaining 37.8% had a family income of over 3000 TD.

The EAT-26, MBSRQ-AS and CAPE-42 scores are shown in table 1.

**Table 1.** The longitudinal evolution of study variables

	Baseline	T 6 months	T 12 months	p	Partial Eta Squared η2
**Disordered eating (EAT-26)**	11.9 ± 9.4	11.9 ± 9.7	12.6 ± 10.2	.080	.006
**Self-classified weight (Body image)**	6.0 ± 1.7	5.9 ± 1.6	6.0 ± 1.6	.946	.001
**Body areas satisfaction**	30.9 ± 6.4	31.8 ± 6.4	31.4 ± 6.8	**.025**	.010
**Overweight preoccupation**	9.3 ± 3.6	9.2 ± 3.7	9.2 ± 3.7	.545	.001
**Appearance Orientation**	41.0 ± 5.4	41.6 ± 5.4	41.6 ± 5.5	**.007**	.014
**CAPE positive dimension (total)**	39.6 ± 8.7	39.1 ± 9.1	39.7 ± 9.5	.756	.001
**Body Mass index**	21.6 ± 3.4	21.5 ± 3.2	21.7 ± 3.2	**.034**	.009

**2) Findings of the mediating analysis**

Disordered Eating scores had no significant effect in subjects across time. However, the effect of the interaction between baseline Overweight Preoccupation with Disordered Eating across time was statistically significant (p=0.036). Overweight Preoccupation (Z=85.095, p<0.001), Body Area Satisfaction (Z=25.053, p<0.001), and CAPE positive dimension (Z=59.931, p<0.001) scores had significant main effects between subjects. (figure 1 and figure 2)

**Image:**

**Figure:**
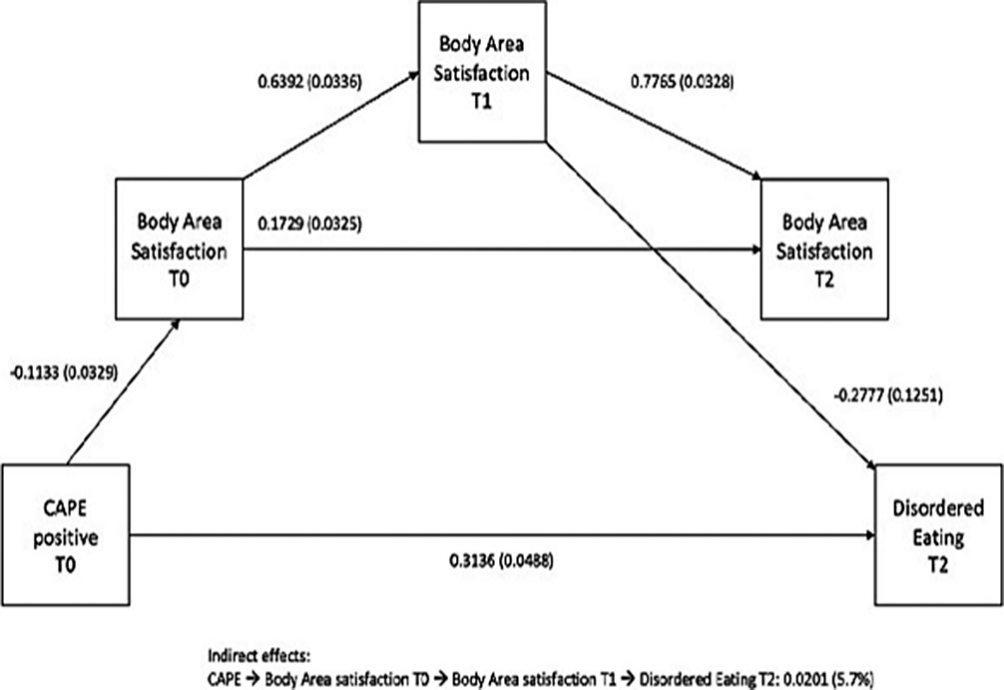

**Image 2:**

**Figure:**
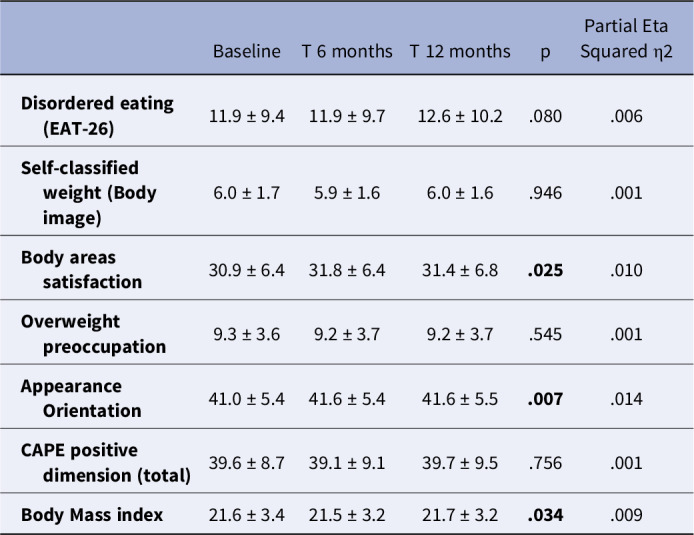

**Conclusions:**

Findings showed that body image disturbances mediated the prospective association between PEs and DE. Adolescents with increased PEs were more likely to experience body image disturbances and, in turn, DE symptoms. These findings offer promising new avenues for prevention and early intervention.

**Disclosure of Interest:**

None Declared

